# Cyclosporin A-Based PROTACs Can Deplete Abundant Cellular Cyclophilin A without Suppressing T Cell Activation

**DOI:** 10.3390/molecules29122779

**Published:** 2024-06-11

**Authors:** Katharina Hilbig, Russell Towers, Marc Schmitz, Martin Bornhäuser, Petra Lennig, Yixin Zhang

**Affiliations:** 1 B CUBE Center for Molecular Bioengineering, Technische Universität Dresden, Tatzberg 41, 01307 Dresden, Germany; hilbig@mpi-cbg.de (K.H.); petralennig@gmx.de (P.L.); 2 Department of Internal Medicine I, University Hospital Carl Gustav Carus, 01307 Dresden, Germany; russell.towers@ukdd.de (R.T.); martin.bornhaeuser@ukdd.de (M.B.); 3 German Cancer Consortium (DKTK), Partner Site Dresden, and German Cancer Research Center (DKFZ), 69120 Heidelberg, Germany; marc.schmitz@tu-dresden.de; 4 National Center for Tumor Diseases (NCT), 01307 Dresden, Germany; 5 School of Cancer and Pharmaceutical Science, King’s College, London WC2R 2LS, UK; 6 Cluster of Excellence Physics of Life, Technische Universität Dresden, 01307 Dresden, Germany

**Keywords:** cyclophilin A (CypA), cyclosporin A (CsA), protein degradation, protein–protein interactions, proteolysis targeting chimera (PROTAC)

## Abstract

Cyclophilin A (CypA), the cellular receptor of the immunosuppressant cyclosporin A (CsA), is an abundant cytosolic protein and is involved in a variety of diseases. For example, CypA supports cancer proliferation and mediates viral infections, such as the human immunodeficiency virus 1 (HIV-1). Here, we present the design of PROTAC (proteolysis targeting chimera) compounds against CypA to induce its intracellular proteolysis and to investigate their effect on immune cells. Interestingly, upon connecting to E3 ligase ligands, both peptide-based low-affinity binders and CsA-based high-affinity binders can degrade CypA at nM concentration in HeLa cells and fibroblast cells. As the immunosuppressive effect of CsA is not directly associated with the binding of CsA to CypA but the inhibition of phosphatase calcineurin by the CypA:CsA complex, we investigated whether a CsA-based PROTAC compound could induce CypA degradation without affecting the activation of immune cells. P3, the most efficient PROTAC compound discovered from this study, could deplete CypA in lymphocytes without affecting cell proliferation and cytokine production. This work demonstrates the feasibility of the PROTAC approach in depleting the abundant cellular protein CypA at low drug dosage without affecting immune cells, allowing us to investigate the potential therapeutic effects associated with the endogenous protein in the future.

## 1. Introduction

The peptidyl-prolyl *cis*/*trans* isomerase (PPIase) CypA is an abundant protein that accounts for 0.1–0.6% of cytoplasmic proteins and is involved in various biological processes [1,2], ranging from protein folding [3] and cellular signaling [4] to mediating the pathology of many different viruses like facilitating the viral life cycle of the human immunodeficiency virus 1 (HIV-1) [5,6] and positively regulating the hepatitis C virus (HCV) replication [7]. It has been identified as the cellular receptor of the immunosuppressive drug CsA [8], which blocks the nuclear factor of activated T cell (NFAT) signaling through inhibition of the Ca^2+^/calmodulin-dependent phosphatase calcineurin (CaN) [9,10,11,12] by the CypA:CsA complex, in turn down-regulating the transcription of cytokine genes such as interleukin-2 (IL-2) and interferon-γ (IFN-γ) [13]. Since CypA contributes to a wide range of diseases including cancers [14], inflammation [15] as in the case of atherosclerosis [16], neurodegenerative diseases [17] like Alzheimer’s disease [17], and viral infections [18,19,20], it represents a valuable drug target [21]. Many non-immunosuppressive CsA derivatives have been reported, while non-immunosuppression means that a compound can inhibit CypA without affecting the calcineurin-NFAT pathway and immune signaling [22]. However, high drug concentrations and doses would be required to inhibit an abundant endogenous protein and exert a therapeutic effect. This in turn could cause undesired side effects. For instance, the treatment of HCV patients with a high dosage of non-immunosuppressive CsA derivative can block virus replication but also leads to developed resistance, the failure of multi-direct-acting anti-viral treatments [23], and causes serious adverse effects such as abdominal pain, fever, and vomiting [23,24].

**Figure 1:**
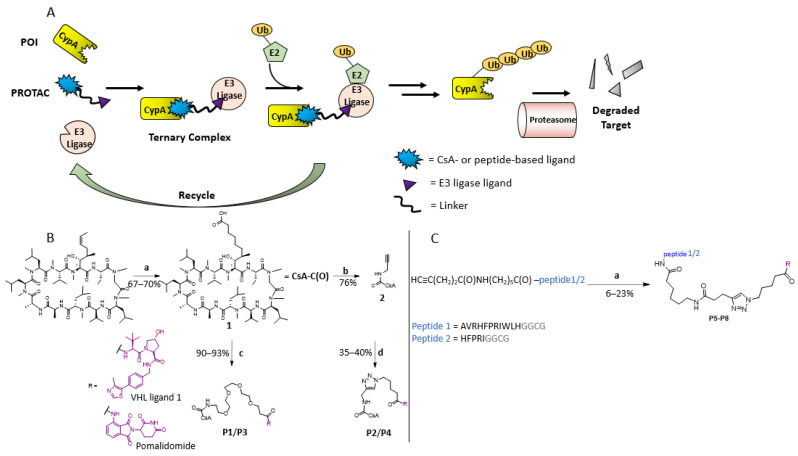
**Design and synthesis of PROTACs against CypA**. (**A**) *Catalytic cycle of the PROTAC technique.* A PROTAC molecule is a bifunctional molecule and consists of a POI ligand, a linker, and an E3 ligase ligand. PROTAC brings the E3 ligase and the POI into proximity, leading to the subsequent degradation of POI by proteasome. In principle, the approach can be realized by using either a high-affinity binder (e.g., **P3**) or a low-affinity binder (e.g., **P5**). (**B**) *Chemical synthesis of CsA-derivative containing a terminal carboxylic group in the side chain of residue 1 and PROTAC compounds* **P1** *to* **P4**. (a) DCM, 0.2 eq. Grubbs Hoveyda II catalyst, 9.0 eq. pent-4-enoic acid, rt, and o.n. 9:1 MeOH/H_2_O, 10% Pd/C, H_2_, rt for 2 h. (b) DMF, 1.1 eq. PEG-linker:E3 ligase ligand compound, 1.2 eq. HATU, 6.0 eq. DIPEA, rt, and o.n. R corresponds to the VHL ligand or pomalidomide. (c) DMF, 1.1 eq. propargylamine, 1.2 eq. HATU, 6.0 eq. DIPEA, rt, and o.n.. R corresponds to the VHL ligand or pomalidomide. (d) Azide-conjugated VHL ligand or pomalidomide, 1:1 DMF/DMSO, 2.0 eq sodium ascorbate, 1.0 eq. CuSO_4_ · 5 H_2_O, 0 °C → 60 °C and o.n.. (**C**) *Chemical synthesis of peptide-PROTACs containing a CypA binding site* **P5** *to* **P8**. (a) Azide-conjugated VHL ligand or pomalidomide, DMSO, 4.0 eq sodium ascorbate, 2.0 eq. CuSO_4_ · 5 H_2_O.

Different from conventional drug molecules, PROTACs (Proteolysis Targeting Chimeras) have the advantage of being organic compounds with catalytic functions [25]. They hijack the activity of the cell’s natural protein degradation machinery, allowing for the deactivation of multiple target molecules with one PROTAC molecule [26] (Figure 1A). PROTACs are heterobifunctional molecules, consisting of (1) the targeting warhead, which binds the protein of interest (POI), (2) the PROTAC anchor, which recruits the E3 ubiquitin ligase, and (3) a linker connecting the two binding moieties [27,28,29]. The formation of a ternary complex (TC) POI:PROTAC:E3 ligase is required for the ubiquitination of the POI by E3 ligase followed by its degradation by the proteasome [26,28,29]. During this process, the PROTAC itself remains intact, resulting in the degradation of multiple POI copies. As PROTACs can function sub-stoichiometrically, they can be used at low concentrations [30]. Therefore, developing PROTAC compounds represents an avenue particularly attractive for inhibiting targets with high cellular concentrations such as CypA [31,32,33]. Moreover, PROTAC represents an “event-driven” pharmacological mechanism rather than an occupation-driven effect [34]. Therefore, different from conventional small molecule inhibitors, PROTAC can antagonize targets by binding to either a functional or allosteric site, while high-binding affinity to POI is also not a prerequisite.

In this work, we have designed PROTAC molecules using either high-affinity CypA binders (CsA derivative) [9] or low-affinity binders (two peptides derived from HIV peptide Vrp) [35] to investigate whether they can deplete this highly abundant cellular protein. This approach can provide the opportunity to directly evaluate the contribution of ligand-binding affinity to the targeted proteolysis. Moreover, since we used an immunosuppressive compound as a warhead to target pathways not related to immunosuppressive treatments, immunosuppression would be considered an adverse effect in this context. Therefore, the CsA-based compounds were further studied regarding their capability to induce cell death and impair T-cell proliferation and cytokine production.

## 2. Results

The CsA derivative with a carboxylic group at residue 1 (compound **1**) was synthesized according to previous reports [36] (Figure 1B). Compound **2** was synthesized by coupling **1** to propargylamine. Compounds **1** and **2** were connected to either VHL E3 ligase ligand (VHL ligand) or CRBN E3 ligase ligand (pomalidomide) using amide bond formation and Cu(I)-catalyzed click reaction, respectively, resulting in PROTAC compounds **P1**–**P4** (Figure 1B and Table 1). While CsA and its derivatives have low nM affinity to CypA [36], the two CypA binding peptides derived from the virus-associated multifunctional protein R of HIV have high µM to low mM affinity to CypA [37]. The peptides containing an alkyne group at their N-terminals were synthesized using solid phase peptide synthesis and conjugated to VHL ligand and pomalidomide with click reaction (Figure 1C, Supporting Information, Figure S1), resulting in the PROTACs **P5**–**P8**.

The PPIase activity assay was used to measure the interaction between PROTACs and CypA (Table 1). As expected, the CsA-based PROTACs exhibited low nM IC_50_ against the PPIase activity of CypA, whereas less than 20% inhibition could be measured for the peptide-based PROTACs at the concentration of 3.3 µM.

**Figure 2:**
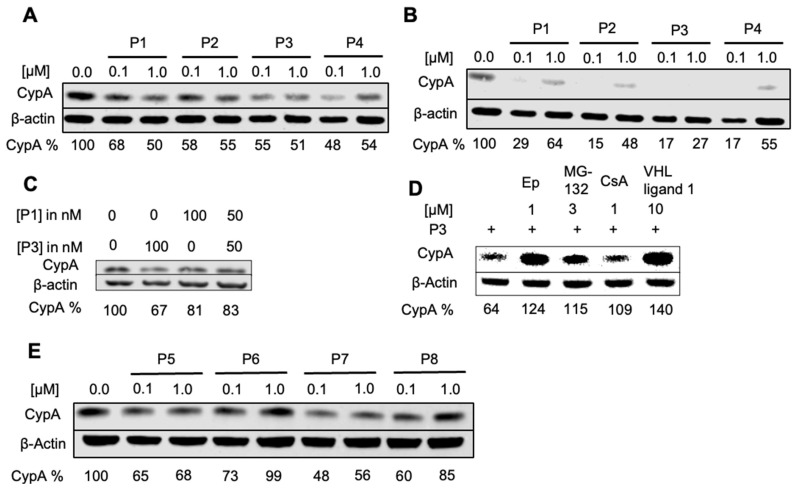
**PROTAC compounds induce CypA degradation in HeLa cells analyzed by western blot.** (**A**) Eight-hour treatment, (**B**) 24 h treatment. (**C**) Treatment for eight hours with a mixture of **P1** and **P3**. (**D**) HDFn were pre-treated for one hour with 1 μM epoxomicin, 3 μM MG-132, 1 μM CsA, and 10 μM VHL ligand 1 and afterward incubated for seven hours with 1 μM **P3**. (**E**) HeLa cells treated for eight hours with peptide-based PROTACs **P5** to **P8**.

The degradation of endogenous CypA in HeLa cells was investigated with 0.1 μM and 1.0 μM CsA-based PROTAC compounds **P1**–**P4** (Figure 2A and Supporting Information Figure S2A) treatments for different durations before cell lysis and western blotting. Remarkable decreases in the intensity of the CypA band were observed after an incubation time of 8 h but not after 4 h. CsA did not affect the amount of CypA in the cell, indicating that the degradation of CypA in the HeLa cell is mediated through the PROTAC mechanism (Figure 2D, Supporting Information Figure S2A). Increasing the treatment time to 24 h further enhanced the degradation, leading to full depletion of cellular CypA (Dmax). Interestingly, a trend was observed where a lower PROTAC concentration (0.1 μM) caused a stronger degradation after 24 h (Figure 2B). This observation can be explained by the “Hook effect”, which predicts a high probability of forming the ternary complex at low PROTAC concentrations [39,40], a characteristic feature for many bi-functional molecules.

The cellular uptake of PROTAC compounds was quantified by mass spectrometry after 4, 8, and 24 h of treating HeLa cells with 100 nM or 1 µM of **P3** or **P4**. Four to twelve-fold higher cellular concentrations were measured with 1 µM of **P3** or **P4** compared to those with 100 nM (Supporting Information, Figure S2B), while the differences between the two concentrations were the largest at 8 h.

Overall, **P3** was revealed as the most efficient PROTAC compound from the experiments, and 70 nM of **P3** was found as the optimal concentration when measuring the degradation of CypA after 6 h of PROTAC treatment (supporting information, Figure S2C,D). VHL ligand coupled-PROTACs (**P3** and **P4**) may have greater stability within the cells [41], and the higher flexibility of the PEG linker (**P3**) [42] compared to the sterically demanding triazole (**P4**) seems to favor the formation of a productive tertiary complex.

As **P1** and **P3** were designed to recruit two different E3 ligases by linking CsA to pomalidomide and VHL ligand, respectively, we investigated whether mixing **P3** and **P1** at 50 nM of each would cause a synergetic effect. As shown in Figure 2C, the combination is less effective than the treatment, with 100 nM of **P3** indicating that the degradation efficiency cannot be augmented by recruiting two different E3 ligases. The PROTAC compounds were also tested in HDFn (Supporting Information, Figure S3A–C), showing **P3** and **P4** as the best PROTACs in fibroblasts, whereas CsA exhibited little effect on CypA degradation in the fibroblasts.

The peptide-based PROTAC compounds (**P5** to **P8**) could also induce the degradation of cellular CypA in HeLa cells and fibroblasts (Figure 2E, Supporting Information Figures S2 and S3). As the peptide-based compounds could be sensitive to the proteolytic activity in cells, we tested their effects after 8 h of treatment. Interestingly, the “Hook effect” was also observed for these compounds. **P5** was found to have an optimal concentration of 50 nM (Supporting Information, Figure S2E).

To demonstrate that the effects of the PROTACs are mediated by the proteasome, HeLa and HDF cells were treated with epoxomicin and MG-132, proteasome inhibitors, before the treatment with **P3** or **P5**. The degradation of CypA in both HeLa cells and fibroblasts was suppressed upon proteasome inhibition (Figure 2D and Supporting Information, Figure S3D,E), confirming the proteasome-mediated degradation mechanism. To further investigate the mechanism of **P3**, the cells were pretreated with VHL ligand 1 or CsA. The resulting recovery of CypA band proves that CypA degradation is indeed induced by **P3** and mediated by its interactions with the POI and with the E3 ligase (Figure 2D and Figure S3E).

**Figure 3:**
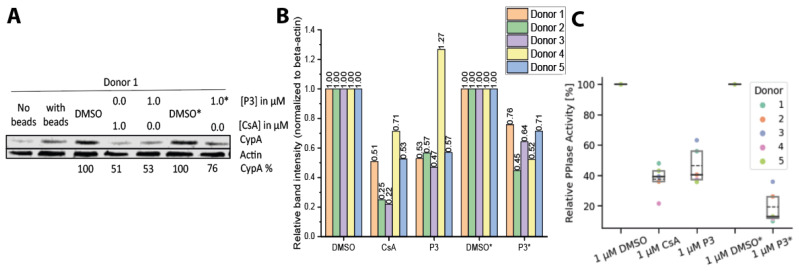
**P3 induces CypA degradation in PBMCs.** (**A**) Western blot of PBMCs from donor one treated with **P3** or CsA after four days or a daily treatment with DMSO or 1 μM **P3** for four days (marked with *). (**B**) Overview of the same experiments as in A) with five different PBMC donors. (**C**) PPIase activity assay of the cell lysate of PBMCs treated with **P3** or CsA for four days, or daily treatment with DMSO or 1 3M **P3** for four days (marked with *). Dashed line marks the mean of the five donors, and the solid line is the median.

To assess the immunosuppressive potential of cellular CypA degradation induced by **P3**, CD3/CD28-activated primary peripheral blood mononuclear cells (PBMCs) from five healthy donors were treated with either **P3** or CsA. PBMCs are commonly used to evaluate the immune activity upon immunosuppressive treatments. CypA protein levels were evaluated by western blot (Figure 3A, donor 1 shown as representative for the five donors, Figure 3B). As expected, **P3** induced the degradation of CypA in the activated PBMCs. CsA exhibited a similar effect, which was not observed in HeLa cells and fibroblasts. While CsA inhibits its immunological target CaN by forming the CypA:CsA:CaN complex, it further acts as molecular glue inducing a new interaction between CypA and an E3 ligase in the immune cells [43,44]. The resulting CypA:CsA:E3 ligase complex could induce the degradation of CypA [45]. The E3 ligase involved in this process remains to be investigated.

We then evaluated the effect of daily administration of 1 µM **P3** for four days. Interestingly, the repeated administration did not cause further degradation of the protein, likely due to the “Hook” effect. Nevertheless, although the higher drug dosage did not cause additional protein degradation, a further reduction in cytosolic PPIase activity was measured (Figure 3C).

**Figure 4:**
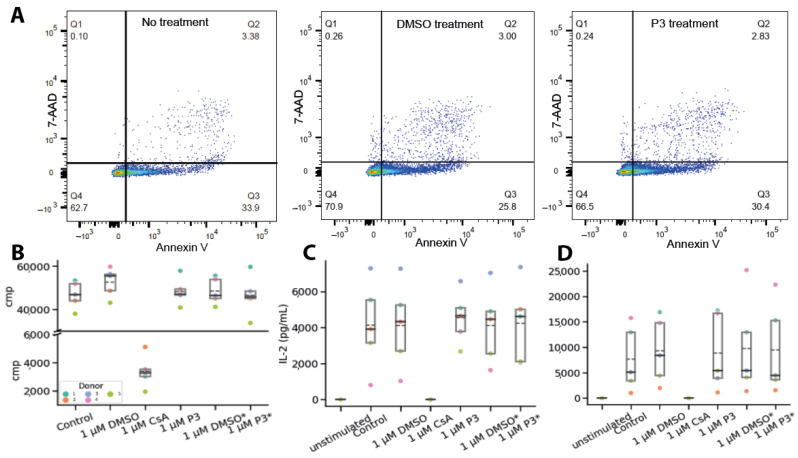
**P3 does not induce cell death or cytokine secretion of PBMCs.** (**A**) FACS experiments to investigate the effect of the PROTAC on PBMC proliferation regarding the necrosis (*y*-axis) and apoptosis (*x*-axis). The markers used to determine the two types of cell death are 7-AAD (necrosis) and Annexin V (apoptosis). A representative dataset of one donor represents two biological replicates/donors. (**A**) PBMCs without treatment and treated with **P3** or DMSO control. (**B**) Proliferation assay with ^3^H-Thymidine. * Daily treatment with DMSO/1 μM **P3** for 4 days. (**C**) ELISA assay with primary antibody against IL-2. Experiments were performed with five different donors. The control defines the IL-2 response of CD3/CD28-stimulated PBMCs. Treatment with DMSO, CsA, and **P3**. * Daily treatment with DMSO or 1 µM **P3** for 4 days. (**D**) ELISA assay with primary antibody against INF-γ. Experiments were performed with five different donors. The control defines the IFN-γ response of CD3/CD28-stimulated PBMCs. Treatment with DMSO, CsA, and **P3**. * Daily treatment with DMSO or 1 μM **P3**. The dashed line marks the mean of the five donors, and the solid line is the median.

The effect of **P3** on the proliferation of PBMCs was determined by FACS measurements, using annexin V and 7-AAD as markers for apoptosis and necrosis, respectively. After treating the cells daily with 1 µM **P3** for four days, there was almost no difference in the resulting percentage of apoptotic and necrotic cells, as compared to the DMSO control (Figure 4A), demonstrating that **P3** does not induce cell death.

The anti-proliferative effect of **P3** on the activated PBMCs was measured by ^3^H thymidine incorporation assay (Figure 4B), with CsA used as a positive control. Neither a single treatment with 1 µM **P3** nor the daily treatment with 1 µM **P3** for four days affected the proliferation of PBMCs, whereas a single treatment with 1 µM CsA led to full inhibition of PBMC proliferation.

The effect of **P3** on important functional properties of the T cells was further evaluated by determining the secretion of cytokines IFN-γ and IL-2 using ELISA. As expected, treating the cells with CsA resulted in fully inhibited production of both cytokines. In contrast, neither a single treatment with 1 µM **P3** nor the daily treatment with 1 µM **P3** for four days affected the production of IFN-γ and IL-2 in activated PBMCs (Figure 4C,D). The observations were consistent between PBMC donors. Therefore, **P3** can induce the degradation of cellular CypA without any detectable immunosuppressive effect in vitro.

## 3. Discussion

Unlike the protein targets of other PROTAC compounds, CypA is a highly abundant cellular protein. It is a PPIase, a large family of enzymes found in both prokaryotes and eukaryotes. Intriguingly, despite their abundance and highly conserved sequence throughout evolution from bacteria and yeast to man, the knockout of all 12 PPIase genes in yeast, including CypA, exhibit no phenotype [46]. CypA-knockout mice also appeared robust and suffered no obvious decrease in life span, while only some animals spontaneously developed allergic diseases [47]. Although CypA is not essential in mammals, it is associated with many viral infections, including HIV [5,48,49,50,51], HCV [7,52,53,54], and SARS-CoV [55,56]. For example, decreased replication of HIV-1 has been found in CypA-knockout T cells [51] as well as in CsA treatment [57]. CypA (encoded by the *PPIA* gene) has also been found overexpressed in many types of cancers and may function as a protective resistance gene in malignant cells [58]. Targeting a highly conserved and endogenous factor, such as CypA, represents a drug discovery approach particularly attractive for antiviral treatment, as it is less likely affected by mutations. As a consequence, many non-immunosuppressive CypA inhibitors have been developed, including non-immunosuppressive derivatives of CsA, derivatives of another immunosuppressive macrocycle, Sanglifehrin, whose structure is not related to CsA, as well as de novo-designed small molecules [22]. Although many of these compounds possess very high affinity (pM to low nM) to CypA, this does not necessarily lead to reduced dosage in the treatment, due to the high cellular concentration of CypA. For example, in phase II clinical studies, the treatment of HCV patients with a high dosage of the non-immunosuppressive CsA derivative (Alisporivir, a low nM CypA inhibitor) can block virus replication, but it also causes serious adverse effects and developed resistance for the patients [23,24].

PROTAC molecules hijack the activity of the cell’s natural protein degradation machinery, allowing for the deactivation of multiple target molecules per drug molecule. They function sub-stoichiometrically and can be used at low concentrations. In this work, we designed and synthesized PROTAC compounds using peptide-based weak binders and CsA-based strong binders. Upon conjugating to VHL ligand or pomalidomide, the resulting PROTAC compounds depleted cellular CypA in HeLa cells, fibroblast cells, and PBMCs. **P3** neither induced cell death nor affected cytokine secretion. Such PROTAC compounds can become powerful pharmacological tools to investigate the elusive pathophysiological functions of this highly abundant and conserved cellular protein and to explore its therapeutic use against viral infections and cancers.

## 4. Materials and Methods

All purified compounds and proteins were analyzed on a reverse-phase Ultra HPLC (Waters ACQUITY UPLC) system with ACQUITY TQ Detector (Waters Corporation, Milford, MA, USA) equipped with an analytical C18 column.

The purification of the compounds was performed by the HPLC (Waters e2698 Separation Module) system using water (0.1%TFA) and acetonitrile (0.1%) as a solvent system with a PDA Detector equipped with a preparative C18(2) column.

### 4.1. Synthesis of E3 Ligase Ligands

VHL ligand, (*S*,*R*,*S*)-AHPC alias VH032, was synthesized starting from 4-bromo-bezylamine according to the guidelines of Steinebach et al. [59]. Pomalidomide was synthesized corresponding to the protocol of Ruchelman et al. [60].

### 4.2. Synthesis of Linker:E3 Ligase Compounds

#### 4.2.1. Compound **7**

To a stirred solution of 2,2-dimethyl-4-oxo-3,8,11,14,17-pentaoxa-5-azaicosan-20-oic acid (31.89 mg, 0.087 mmol, 1.0 eq.) and compound **6** (52.24 mg, 0.096 mmol, 1.1 eq.) in 0.21 mL DMF, HATU (39.70 mg, 0.104 mmol, 1.2 eq.) and DIPEA (90.93 μL, 0.522 mmol, 6.0 eq.) were added. The mixture was stirred overnight at room temperature and was afterward quenched by the addition of water. The aqueous layer was three times extracted with EtOAc. The combined organic layers were washed with 5% (*w*/*v*) citric acid as well as NaHCO_3_ solution and dried over anhydrous Na_2_SO_4_. After evaporation of the solvent by reduced pressure, the product (67.0 mg, 0.085 mmol, 98%) was purified by HPLC. Compound **7** was obtained through the deprotection by 20% TFA in DCM.

Yield: 98%, LC-MS: *m*/*z*: 678.10 (M–H)^+^

#### 4.2.2. Compound **11**

DMF (0.185 μL) was added at room temperature to a stirred solution of 2,2-dimethyl-4-oxo-3,8,11,14,17-pentaoxa-5-azaicosan-20-oic acid (Thermo Fisher, Waltham, MA, USA, 30.0 mg, 0.082 mmol, 2.23 eq.) and oxalyl chloride (VWR, Radnor, PA, USA, 6.35 μL, 0.074 mmol, 2.0 eq.) in 50.0 μL diethyl ether (VWR). After 3 h, 4-amino-2-(2,6-dioxopiperidin-3-yl)isoindoline-1,3-dione **10** (10.0 mg, 0.037 mmol, 1.0 eq.) in THF (VWR, 370.0 μL) was added to the mixture and heated to reflux (70 °C) overnight. To the mixture, water was added, and the aqueous layer was extracted with DCM (dichloromethane) (VWR). The combined organic layers were dried over anhydrous Na_2_SO_4_. After evaporating the solvents by vacuum, the title compound was observed and purified through HPLC. The deprotection by 20% TFA in DCM for 30 min at room temperature led to product **11** (44.4 mg, 0.07 mmol).

LC-MS: *m*/*z*: 520.92 (M–H)^+^

#### 4.2.3. Compound **12**

DMF (0.17 μL) was added at room temperature to a stirred solution of 5-bromopentanoic acid (Sigma Aldrich, St. Louis, MO, USA, 8.88 mg, 0.049 mmol, 2.23 eq.) and oxalyl chloride (3.77 μL, 0.044 mmol, 2.0 eq.) in 30.0 μL diethyl ether. After 3 h, 4-amino-2-(2,6-dioxopiperidin-3-yl)isoindoline-1,3-dione **10** (6.01 mg, 0.022 mmol, 1.0 eq.) in THF (220.0 μL) was added to the mixture and heated to reflux (80 °C) overnight. Water was added to the mixture, and the aqueous layer was extracted with DCM. The combined organic layers were dried over anhydrous Na_2_SO_4_. The yellow residue **12** (19.5 mg, 0.045 mmol) was utilized for the next step without further purification.

Yield: 85%, LC-MS: *m*/*z*: 437.75 (M–H)^+^

#### 4.2.4. Compound **13**

An amount of 5.82 mg NaN_3_ (Merk, Darmstadt, Germany, 0.089 mmol, 2.0 eq.) was added to a stirred solution of 4-bromo-*N*-(2-(2,6-dioxopiperidin-3-yl)-1,3-dioxoisoindolin-4-yl)butanamide **12** (19.5 mg, 0.045 mmol, 1.0 eq.) in 89.4 μL dry DMF. The mixture was heated to 80 °C overnight. After cooling down to room temperature, the reaction mixture was extracted with EtOAc. The organic layers were concentrated by vacuum to obtain the yellow solid **13** (12.0 mg, 0.03 mmol, 67%). The product was used without further purification.

Yield: 67%, LC-MS: *m*/*z*: 399.02 (M–H)^+^

#### 4.2.5. Compound **15**

HATU (7.3 g, 0.019 mmol, 1.2 eq.) and DIPEA 16.7 μL, 0.087 mmol, 6.0 eq.) were added to a solution of compound **14** (2.29 mg, 0.014 mmol, 1.0 eq.) and compound **6** (8.7 mg, 0.016 mmol, 1.1 eq.) in 100.0 μL DMF. The mixture was stirred overnight at room temperature and was afterward quenched by the addition of water. The aqueous layer was three times extracted with EtOAc. The combined organic layers were dried over anhydrous Na_2_SO_4_. After evaporation of the solvent by reduced pressure, the product **15** (6.66 mg, 0.012 mmol, 86%) was obtained.

Yield: 86%, LC-MS: *m*/*z*: 555.98 (M–H)^+^

### 4.3. Synthesis of CsA-Based PROTACs

CsA (LC Laboratories, Woburn, MA, USA, 50.0 mg, 0.042 mmol, 1.0 eq.) was activated in 0.5 mL DCM with 5.26 mg Grubbs Hoveyda II catalyst (Sigma Aldrich, 0.008 mmol, 0.2 eq.) for 30 min at room temperature. After adding pent-4-eonic acid (Alfa Aesar, Haverhill, MA, USA, 38.21 μL, 0.374 mmol, 9.0 eq.), the mixture was heated to 45 °C overnight. The solution was filtered over silica in 9:1 EtOAc/MeOH to obtain 72.0 mg (0.057 mmol) of the product.

LC-MS: *m*/*z* = 1260.82

The compound (72.0 mg, 0.057 mmol, 1.0 eq.) was stirred in 1.5 mL 9:1 MeOH/H_2_O with a spade point of Pd/C (10%) for 2 h under H_2_ atmosphere. Afterward, the mixture was filtered over celite (VWR), and the red solid **1** (82.03 mg, 0.065 mmol) was utilized without further purification.

LC-MS: *m*/*z*: 1262.55

The general procedure followed for amide bond formation with compound **1** (CsA–COOH) was:

HATU (1.2 eq.) was added to a solution of compound **1** (1.0 eq.) in DMF and stirred for 30 min at room temperature. Then, the mixture was completed with the amine compound (1.1 eq.) and DIPEA (6.0 eq.) and stirred overnight at room temperature. The reaction was quenched by the addition of water, and the aqueous layer was three times extracted with EtOAc. The combined organic layers were dried over Na_2_SO_4_. The solvent was evaporated through a rotary evaporator to obtain the product.

#### 4.3.1. Compound **2**

Propargylamine was used as an amine compound to lead to product **2**.

Yield: 60%, LC-MS: *m*/*z*: 1299.31 (M–H)^+^

#### 4.3.2. Compound **P1**

Yield: 90%, LC-MS: *m*/*z*: 1765.05

#### 4.3.3. Compound **P3**

Yield: 16%, LC-MS: *m*/*z*: 1922.31

### 4.4. General Procedure for the Click Reaction with Compound ***4***

An aqueous solution of CuSO_4_∙5 H_2_O (Merck, 1.0 eq.) and sodium ascorbate (Alfa Aesar, 2.0 eq.) prepared on ice was quickly added to a stirred solution of compound **4** (1.0 eq.) and the corresponding azide (1.0 eq.) in 1:1 DMF/DMSO (Dimethyl sulfoxide, VWR). The mixture was heated to 60 °C and stirred overnight. Then, the reaction was quenched by the addition of water. The aqueous layer was three times extracted with EtOAc. After the solvent was evaporated, the title compound was yielded and purified through HPLC.

#### 4.4.1. Compound **P2**

Yield: 34%, LC-MS: *m*/*z*: 1697.88

#### 4.4.2. Compound **P4**

Yield: 36%, LC-MS: *m*/*z*: 1855.06

### 4.5. Synthesis of Peptide-Based PROTACs

The following peptides AVRHFPRIWLHGGCG and HFPRIGGCG (50 μmol) were synthesized at the Liberty Blue of CEM with 1.0 eq. of DIC (*N*,*N*-Diisopropylcarbodiimide, Iris Biotech, Marktredwitz, Germany) and 0.5 eq. of OximaPure (Ethyl cyano(hydroxyimino), Iris Biotech). The amino acids used were purchased from Iris Biotech.

AVRHFPRIWLHGGCG: LC-MS: *m*/*z*: 1705.03

HFPRIGGCG: LC-MS: *m*/*z*: 942.31

For further modification, the resin was soaked in DMF and the acid component 6-(Fmoc-amino) hexanoic acid (Iris Biotech, 53.0 mg, 0.15 mmol, 3.0 eq.) was activated with HATU (57.0 mg, 0.15 mmol, 3.0 eq) in 1.5 mL DMF for 45 min. After the addition of DIPEA (78.39 μL, 0.45 mmol, 9.0 eq.), the reaction mixture was added to the resin and constantly shaken overnight. Afterward, the reaction mixture was discarded, and the resin was washed three times each with DMF and DCM.

AHX-AVRHFPRIWLHGGCG: LC-MS: *m*/*z*: 1020.79 (M–2H)^2+^

AHX-HFPRIGGCG: LC-MS: *m*/*z*: 1277.54

Additionally, the resin was soaked in DMF and the acid 4-pentyoinic acid (Sigma Aldrich, 14.72 mg, 0.15 mmol, 3.0 eq.) was activated with HATU (57.0 mg, 0.15 mmol, 3.0 eq) in 1.5 mL DMF for 45 min. After the addition of DIPEA (78.39 μL, 0.45 mmol, 9.0 eq.) the reaction mixture was added to the resin and constantly shaken overnight. Afterward, the reaction mixture was discarded, and the resin was washed three times each with DMF and DCM.

Pent-AHX-AVRHFPRIWLHGGCG: LC-MS: *m*/*z*: 1060.54 (M–2H)^2+^

Pent-AHX-HFPRIGGCG: LC-MS: *m*/*z*: 1357.64

An aqueous solution of CuSO_4_∙5 H_2_O (Merck, Darmstadt, Germany, 1.0 eq.) and sodium ascorbate (Alfa Aesar, 2.0 eq.) prepared on ice was quickly added to a stirred solution of ether modified peptide (1.0 eq.) and the corresponding azide (1.0 eq.) in 1:1 DMF/DMSO (Dimethyl sulfoxide, VWR). The mixture was heated to 60 °C and stirred overnight. Then the reaction was quenched by the addition of water. The aqueous layer was three times extracted with EtOAc. After the solvent was evaporated, the title compound was yielded and purified through HPLC.

#### 4.5.1. Compound **P5**

Pent-AHX-AVRHFPRIWLHGGCG

Yield: 23%, LC-MS: *m*/*z*: 1148.32 (M–2H)^2+^

#### 4.5.2. Compound **P6**

Pent-AHX-HFPRIGGCG

Yield: 10%, LC-MS: *m*/*z*: 1533.64

#### 4.5.3. Compound **P7**

Pent-AHX-AVRHFPRIWLHGGCG

Yield: 19%, LC-MS: *m*/*z*: 1227.45 (M–2H)^2+^

#### 4.5.4. Compound **P8**

Pent-AHX-HFPRIGGCG

Yield: 6%, LC-MS: *m*/*z*: 1690.32

### 4.6. Cell Culture

HeLa cells and primary human dermal fibroblasts isolated from neonatal foreskin (HDFn) were adherent cells and were cultured in commercially available, ready-to-use DMEM (Dulbecco’s minimal essential medium, Gibco, Waltham, MA, USA) supplemented with 10% (*v*/*v*) FBS (Fetal Bovine Serum, Sigma-Aldrich) and antibiotics (Streptomycin/Penicillin, 1:1000 dilution) in polystyrene culture flasks. Growth conditions for all cell lines were maintained in a humidified chamber at 37 °C and 5% CO_2_. For subculturing or prior counting, the cells were treated with trypsin-EDTA (Life Technologies GmbH, Carlsbad, CA, USA) to detach them from the surface.

A total of 5 × 10^5^ HeLa cells or HDFn was plated in 6-well plates the day before treatment. In the case of epoxomicin (Sigma-Aldrich), MG-132 (Sigma-Aldrich), CsA, (LC Laboratories), or VHL ligand (Broad Pharm, San Diego, CA, USA) treatment, they were dosed with 1 μM epoxomicin, 3 μM MG-132, 1 μM CsA or 10 μM VHL ligand in DMSO (100 μL) for 1 h. An amount of 200 μL PROTAC dilutions (0.1 μM and 1.0 μM) in DMSO (final concentration 0.1% (*v*/*v*)) were added to the culture medium and incubated at 37.0 °C and 5.0% CO_2_ for the desired time. As a blank, the cells were incubated with 200 μL DMSO under the same conditions. After the appropriate incubation time, the medium was aspirated, and the cells were washed twice with 1× PBS.

### 4.7. PBMC Isolation

Blood was obtained from healthy volunteers after obtaining written consent (Ethical approval No. EK206082008). PBMCs were isolated by density gradient centrifugation using Pancoll (PAN-Biotech, Aidenbach, Germany), washed twice with Dulbecco’s Phosphate Buffered Saline (Gibco, USA), and finally resuspended in Roswell Park Memorial Institute 1640 (Gibco, USA) supplemented with 10% fetal bovine serum (Sigma-Aldrich, USA).

### 4.8. Western Blotting

Cells were lysed in ice-cold 1× Laemmli buffer (100 mM DTT) after two washes with 1× PBS. After denaturation (95 °C for 5 min) and shearing of the DNA by sonication, the samples were loaded on Tricine-SDS-PAGE, electrophoresed, and blotted onto a PVDF membrane. After staining with S-Ponceau staining solution and blocking, the membranes were incubated overnight at 4 °C in primary rabbit anti-CypA antibody (Cell Signaling Technology #2175S, Danvers, MA, USA) and for 1 h at room temperature with secondary antibody (IRDye^®^ 800 CW Goat anti-Rabbit IgG Secondary Antibody, LI-COR). All membranes were then reprobed with rabbit anti-beta actin (Cell Signaling Technology #4967) to ensure equal protein loading. The intensities of the bands on western blot films were quantified using the LI-COR Odyssey Imaging System and the software Image Studio Lite (version 3.1). The CypA values were normalized to β-actin, and DMSO was arbitrarily assigned a value of 100% for comparison purposes.

### 4.9. PPIase Activity Assay

The assay was performed in HEPES buffer (35 mM HEPES, 150 mM NaCl, 1 mM DTT, pH 7.8). Suc-AFPFpNA (Sigma-Aldrich) was dissolved in DMSO to a final concentration of 20 mg mL^−1^ and further diluted in HEPES buffer to a final concentration of 600 μg mL^−1^ (10× the substrate stock). α-Chymotrypsin was prepared in 1 mM HCl with a final concentration of 60 mg mL^−1^. After the termination of proteolysis by 200 μL of 30% acetic acid, the samples were injected into UPLC. Chromatographic separation was performed using a gradient at a flow rate of 0.5 mL/min with mobile phase A consisting of water (0.1% FA) and mobile phase B consisting of acetonitrile (0.1% FA) for 4 min while monitoring a range from 300 to 400 nm. The linear gradient was applied from 5% to 95% of mobile phase B from 0.5 to 3.5 min.

The concentration of the substrate and product (pNA) was calculated by calibration curves. The peak areas of Suc-AFPFpNA (l= 318 nm at 2.44 min) and 4-nitroaniline (pNA, l = 383 nm at 1.60 min) were determined using MassLynx 4.2 (Waters Corporation, Milford, MA, USA) to evaluate PPIase activity by calculation in Origin 2019 64 bit software (OriginLab Corporation, Northampton, MA, USA).

### 4.10. IC_50_ Determination

A 4 nM concentrated CypA was incubated with 0.5 μL of the inhibitor (different concentrations) and the substrate in the buffer for 30 min. Then, 5 μL of chymotrypsin was added to the sample series and stirred rapidly for 30 s. After 120 s, 200 μL of 30% acetic acid was added to the series and pipetted up and down 15 times. The half-maximal inhibitory concentration (IC_50_) was determined by dose–response fitting.

### 4.11. Evaluation of the PPIase Activity of Cellular CypA

A total of 5 × 10^5^ cells was lysed in HEPES buffer by freeze and thaw cycles and 4.75 μL of that cell lysate was added to a mixture of HEPES buffer supplemented with 100 nM SLF to inhibit FKBP, 10× pNA in HEPES/SLF buffer and 0.5 μL DMSO and incubated on ice for 30 min. Then, 5 μL of chymotrypsin was added to the sample series using a multichannel pipette and stirred rapidly for 30 s. After 120 s, 200 μL of 30% acetic acid was added to the series and pipetted up and down 15 times. The samples were injected into the UPLC. In this case, the blank consisted of 79.75 μL HEPES, 4.75 μL lysis buffer, and 10 μL 10 × pNA in HEPES/SLF buffer.

### 4.12. PBMC Toxicity Assessment

A total of 1 × 10^5^ PBMCs was stimulated with anti-CD3/CD28 Dynabeads (Gibco, USA) and cultured in Roswell Park Memorial Institute 1640 (Gibco, USA) supplemented with 10% fetal bovine serum (Sigma-Aldrich, USA) [1 μM DMSO, P3 or CsA and daily treatment with 1 μM DMSO/P3]. After 4 days of incubation at 37 °C/5%CO_2_, cells were collected and assessed for apoptosis by flow cytometry (LSR II, BD, Franklin Lakes, NJ, USA) using the commercial PE-Annexin V Apoptosis Detection Kit (BD, USA).

### 4.13. PBMC Proliferation Assay and Cytokine Assessment

A total of 1 × 10^5^ PBMCs was stimulated with anti-CD3/CD28 Dynabeads (Gibco, USA) and cultured in Roswell Park Memorial Institute 1640 (Gibco, USA) supplemented with 10% fetal bovine serum (Sigma-Aldrich, USA) [1 μM DMSO, P3 or CsA and daily treatment with 1 μM DMSO/P3]. After 3 days of incubation at 37 °C/5%CO_2_, 1 µCi ^3^H-thymidine (Hartmann Analytic, Braunschweig, Germany) was added to the cultures and further incubated for an additional 18 h. Cells were harvested and proliferation was assessed as a measurement of ^3^H-thymidine incorporation as determined with the 1450 MicroBeta TriLux (PerkinElmer, Waltham, MA, USA), converting degrees of radioactivity into counts per minute (cpm). IFNγ and IL-2 assessment of supernatants was performed by commercial enzyme-linked immunosorbent assay kits (BD, USA).

## Figures and Tables

**Table 1:** *Characterization of anti-CypA PROTACs*. These differ in their warhead, E3 ligase ligand, and linker. The dissociation constant (IC_50_) values between the PROTACs and CypA were determined with the PPIase activity assay [38].

	E3 Ligase Warhead	Linker	POI Warhead	IC_50_
**P1**	Pomalidomide	–(–CH_2_–CH_2_–O)_4_–	CsA	7.73 nM
**P2**	Pomalidomide	Triazole	CsA	1.80 nM
**P3**	VHL-ligand	–(–CH_2_–CH_2_–O)_4_–	CsA	13.54 nM
**P4**	VHL-ligand	Triazole	CsA	3.50 nM
**CsA**	-	-	-	5.70 nM
**P5**	Pomalidomide	–(–CH_2_–CH_2_–O)_4_–	Peptide 1	>3 µM
**P6**	Pomalidomide	Triazole	Peptide 2	>3 µM
**P7**	VHL-ligand	–(–CH_2_–CH_2_–O)_4_–	Peptide 1	>3 µM
**P8**	VHL-ligand	Triazole	Peptide 2	>3 µM

## Data Availability

The original contributions presented in this study are included in the article/Supplementary Material; further inquiries can be directed to the corresponding author.

## Supplementary Materials

The following supporting information can be downloaded at: https://www.mdpi.com/article/10.3390/molecules29122779/s1, Figure S1: Synthesis of the Vrp-derived peptide-PROTACs; Figure S2: Testing PROTAC compounds in Hela cells; Figure S3: Western blots of HDFn treated with PROTACs and CsA. Synthesis of Vrp-peptide derived PROTACs and further characterization of CsA-based and peptide-based PROTACs in two cell lines are shown, as well as a proof of the mechanism of degradation by the proteasome. Further we quantify the cellular uptake of PROTAC P3 and P4.
