# Supplementary material for: Cyclosporin A-Based PROTACs Can Deplete Abundant Cellular Cyclophilin A without Suppressing T Cell Activation
Source: Molecules. 2024 Jun 11;29(12):2779. doi: 10.3390/molecules29122779 (PMC11206246; doi:10.3390/molecules29122779)
Supplement: Supplementary file 1 [file molecules-29-02779-s001.zip › molecules-2971978-supplementary.pdf]

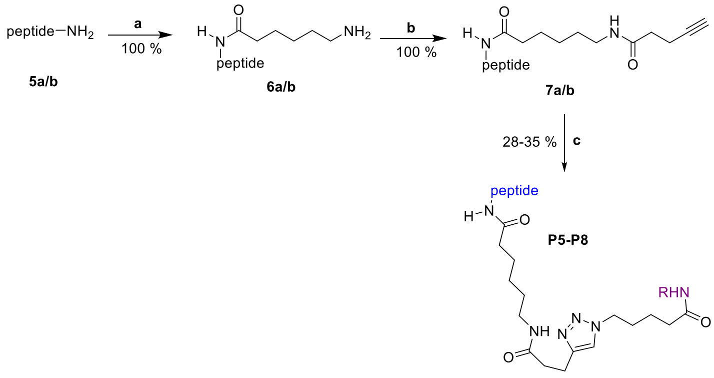

Figure S1: *Synthesis of the Vrp-derived peptide-PROTACs.* **a)** DMF, 3.0 eq. AHX, 3.0 eq. HATU, 6.0 eq. DIPEA, rt, and o.n.. **b)** DMF, 3.0 eq. pent-4-enoic acid, 3.0 eq. HATU, 6.0 eq. DIPEA, rt, and o.n.. **c)** DMSO, 4.0 eq sodium ascorbate, 2.0 eq. $CuSO_4 \cdot H_2O$, 0 °C → rt and o.n.. R corresponds to the VHL ligand or pomalidomide.

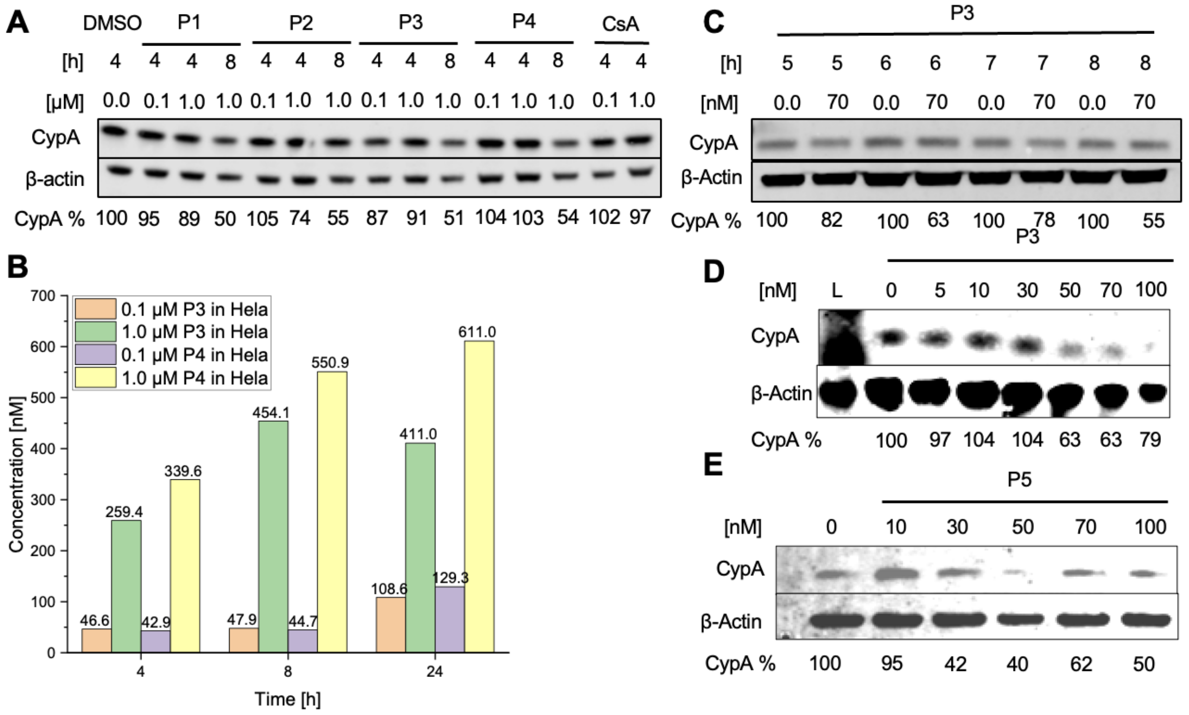

Figure S2: **A)** Western blot of Hela cells treated with the PROTACs **P1** to **P4** and CsA for four hours. **B)** Quantification of cellular PROTAC uptake of **P3** and **P4** of Hela cells treated with the additives for four, eight, and 24 hours. The experiments were done with MS/MS and SLF as internal standards. **C)** Western blot of Hela cells treated with 70 nM of **P3** for different time frames. **D)** Western blot of Hela cells treated for six hours with the PROTACs **P3** in different concentrations ranging from 10 to 100 nM. **E)** Western blot of Hela cells treated for eight hours with the PROTACs **P5** in different concentrations ranging from 10 to 100 nM.

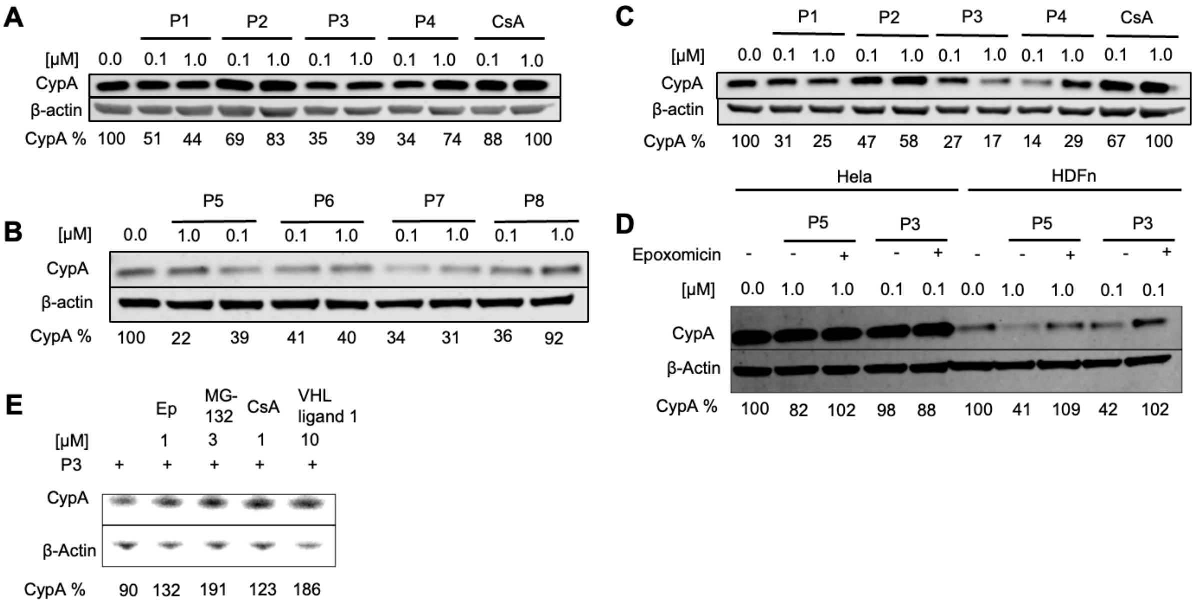

Figure S3: Western blots of HDFn treated with PROTACs and CsA for **A)** eight hours with **P1** to **P4**, **B)** 24 hours with **P1** to **P4**, and **C)** eight hours with **P5** to **P8**. **D)** Hela cells and HDFn pre-treated for one hour with 1 µM epoxomicin and eight hours with **P3** and **P5** **E)** Hela cells pre-treated with epoxomicin, MG-132, CsA, and VHL ligand 1 treated for seven hours with **P3**.
